# Roles of mast cells and their interactions with the trigeminal nerve in migraine headache

**DOI:** 10.1177/17448069231181358

**Published:** 2023-06-02

**Authors:** Leo C Guan, Xinzhong Dong, Dustin P Green

**Affiliations:** 1 141369 McDonogh School, Owings Mills, MD, USA; 2 Department of Neuroscience, Johns Hopkins University, School of Medicine, 60790 Howard Hughes Medical Institute, Baltimore, MD, USA; 3 Department of Neurobiology, 12338 University of Texas Medical Branch, Galveston, TX, USA

**Keywords:** Migraine, mast cells, trigeminal nerve, sensory neuron, neurogenic inflammation

## Abstract

Migraine pain is characterized by an intense, throbbing pain in the head area and possesses complex pathological and physiological origins. Among the various factors believed to contribute to migraine are mast cells (MCs), resident tissue immune cells that are closely associated with pain afferents in the meninges. In this review, we aim to examine and discuss recent findings on the individual roles of MCs and the trigeminal nerve in migraine, as well as the various connections between their mechanisms with an emphasis on the contributions those relationships make to migraine. This is seen through MC release of histamine, among other compounds, and trigeminal nerve release of calcitonin-gene-related-peptide (CGRP) and pituitary adenylate cyclase activating peptide-38 (PACAP-38), which are peptides that are thought to contribute to migraine. Secondly, we illustrate the bi-directional relationship of neurogenic inflammation as well as highlight the role of MCs and their effect on the trigeminal nerve in migraine mechanisms. Lastly, we discuss potential new targets for clinical interventions of MC- and trigeminal nerve-mediated migraine, and present future perspectives of mechanistic and translational research.

## Introduction

Headache is the most common neurological disorder, with migraine ranked third among people aged 15–49 years.^1^ Headache is described as pain in the head, scalp, and/or neck in varying degrees of intensity. Migraine headache, however, is closely associated with severe throbbing pain and is commonly accompanied by symptoms such as nausea, visual and auditory sensitivity, and an array of possibly debilitating effects. Additionally, migraine is characterized by recurrent headaches with a greater intensity than normal headaches. Episodic migraine, defined as 0 to 14 migraines per month, possesses fewer disabling effects in comparison to chronic migraine, which is characterized by over 15 migraines per month.^2^

Migraine with aura is a unique neurological phenomenon that precedes the headache phase, with aura progressing gradually and eliciting sensory, speech, and/or motor symptoms.^3^ Studies have demonstrated that migraine with aura possesses varying heritability, greater association with other diseases, and altered brain structure and function in comparison to migraine without aura.^2^ Additionally, migraine also shows gender differences and may be dependent on sex hormones. For example, migraine is two to three times more common in women than in men.^3^ Furthermore, migraine severity, which is characterized by attack duration, headache intensity, frequency, and occurrence of symptoms, is also found to be greater in women.^3^

The underlying causes of migraine headaches have long been studied and increasing evidence points to the source of migraine as a complex disorder caused by both vascular and neuronal factors.^4^ Since the pathophysiology leading to the generation of migraine includes an array of factors such as vascular changes, nociceptor activation, neuropeptide release, and neurogenic inflammation,^5^ this review aims to focus on these factors and how they may contribute to the generation of migraine. We also outline the role of an important immune cell, mast cells (MCs), and their interactions with trigeminal nerves as co-participants in migraine. Finally, we discussed current migraine treatment, potential new targets for clinical interventions of MC- and trigeminal nerve-mediated migraine, and future perspectives of mechanistic and translational research.

## Methods

The literature used in this review is focused on the potential roles of MCs and their interactions with the trigeminal nerve in migraine. Accordingly, we used keywords including headache, migraine, MCs, trigeminal nerve, and neurogenic inflammation for searching peer-reviewed articles published in recent years in indexed medical journals. We also examined the reference lists of the sources selected to identify additional studies not found in the original search.

## Origins and etiology of migraine

### Vasodilation and role of calcitonin-gene-related-peptide (CGRP) in migraine

Early studies in human patients with the triptan class of 5-HT1 agonists implicated vascular disorders as the underlying cause of migraine.^6,7^ When examining the causes of vasodilation in migraine, multiple pre-clinical studies implicated the role of CGRP in contributing to migraine pathophysiology.^5^ CGRP is a neuropeptide widely recognized as a potent vasodilator,^8^ and is released during the activation of perivascular meningeal nociceptors in the dura mater. The presence of CGRP in meningeal blood vessels was previously hypothesized to result in activation and sensitization of trigeminal meningeal nerve afferents, which is then lead to headache. However, subsequent studies utilizing electrophysiological recordings found that CGRP could not directly excite meningeal afferents, By measuring dural blood flow and recording meningeal nociceptor activation 10–15 min after CGRP administration in rats, Levy et al.^9^ found that CGRP did increase dural blood flow but did not activate meningeal nociceptors. This finding implies that although CGRP plays a role in the vasodilation of dural blood vessels but exerts no effect on meningeal sensitization.

A more recent clinical study examined the role of vasodilation in migraine without the involvement of CGRP,^10^ by using magnetic resonance angiography in patients with cilostazol-induced unilateral migraine. The magnetic resonance angiography scans were performed at various stages throughout the migraine including at baseline, at onset, after sumatriptan, and 27 h after the initial onset of the migraine. The study found that during the onset of migraine, the meningeal artery increased in caliber only on the side of the head where the pain was described by the patient, implying a role of vasodilation in migraine. Authors further suggested that the vasodilation of the middle meningeal artery may be a biomarker for the activation of dural nociceptors, indicating an anatomical site for migraine headaches. These clinical findings imply that even if vasodilation is not the sole cause of migraine, vasodilation still may have an important role in the onset of migraine as it likely participates in more complex mechanisms than those previously understood.

### Role of the trigeminal meningeal nerve in mediating migraine

The trigeminal nerves are one of the largest cranial nerves, and consists of three divisions: the ophthalmic, maxillary, and mandibular divisions which span from the trigeminal ganglion located around the temple on the left and right of the head.^11^ The upper divisions, the ophthalmic and maxillary, provide sensory information while the mandibular division incorporates both motor and sensory information as it is partly responsible for the innervation of the mouth and its components. The center of the left and right of the head possess a trigeminal ganglion which provides and receives information from the three divisions.^11^ Together these afferent nerves are thought to be the primary source of orofacial pain and migraine pain. Intriguingly, trigeminal ganglion neurons widely express both ER_α_ and ER_β_ estrogen receptors, providing a biological basis for the sex difference in migraine prevalence.^3^

Since the trigeminal nerves are responsible for the innervation of most regions of the head and are a source of CGRP, they have been a primary target for migraine research and treatment.^12^ The dura mater of the meninges is densely innervated by the meningeal nerves, a branch of the trigeminal nerve’s ophthalmic division. This introduces the relationship of meningeal blood vessel caliber to the function of the trigeminal meningeal nerve through their near proximity to each other. The trigeminal nerve terminals can release pro-inflammatory neuropeptides substance P (SP) and CGRP.^13^ These compounds, released from intracranial sources such as the trigeminal nerve, travel through venous blood where their various mediating functions and vasodilating effects can then take place.^14–16^ Thus, the trigeminal nerve may hold a significant role in regulating the interactions of cranial systems such as those that contribute to migraine headaches.^14,16^

### Mast cell degranulation and neurogenic inflammation

MCs are versatile immune cells that can respond to a variety of stimuli and are implicated in most physiological and pathological conditions. MCs originate from pluripotent progenitor cells in bone marrow^17^ where they then enter the bloodstream and migrate to various tissues, eventually maturing into resident immune cells.^18^ MCs tend to reside in areas with close contact with the external environment, including sensory nerve endings, allowing MCs to communicate with nociceptors as a responsive presence between the external and internal environments. This important anatomical location of MCs therefore allows them to serve as ‘first responders’ to external and internal stimuli. The primary role of MCs is their participation in immune response through activation and degranulation mechanisms, releasing stored vasoactive compounds that increase vascular permeability, accumulation of fluid, and recruit other immune cells such as eosinophils, natural killer (NK) cells, monocytes, macrophages, and neutrophils, further intensifying and magnifying the inflammatory response.^17,19–21^

Numerous bioactive and immunomodulatory molecules such as chemokines, cytokines, histamine, serotonin, and tryptase are stored in MCs.^22,23^ The most commonly understood mechanism of MC degranulation involves the binding of allergens to immunoglobin E (IgE),^24^ causing cross-linking between IgE and its high-affinity receptor Fc epsilon RI (FcεR1).^25,26^ MCs contain a diverse set of cell-surface receptors which each possess different activation prerequisites, and when undergoing degranulation can release an array of products.^27^ MC activation results in degranulation and the release of compounds such as histamine, serotonin, prostaglandins, tryptase, and cytokines, and elevates other pro-inflammatory mediators such as tumor necrosis factor a (TNFa) and interleukins,^26^ which are capable of both potent inflammation and vasodilation.^26^

## The involvement of meningeal mast cells in migraine

### Meningeal mast cells

Mast cell spatial advantage often makes them the first immune cells to respond to external and internal stimuli.^28^ Meningeal MCs are closely associated with the nociceptors in the dura, and histamine and cytokines released by these resident immune cells are elevated in migraineurs.^29,30^ MCs are most well known for their involvement in allergy, however, they also possess a primary role in pathological and physiological interactions through aforementioned activation and degranulation processes. Thus, MCs are a crucial component of immune defense and regulation, as their activation and degranulation are also responsible for the onset of allergy and its immediate reactions, which migraine is often associated with.

Through their vasodilating, activating, sensitizing, and inflammatory capabilities, MCs have been understood to be a participant in the onset of migraine. In particular, MCs contribute an important portion of serotonin, prostacyclin (PGI_2_), and histamine, which have been identified as capable of eliciting migraine.^31,32^ Historically, histamine has been known for its role in migraine pathogenesis through its binding with the H1 and H2 receptors and recently discovered H3 and H4 receptors. H1 and H2 receptor antagonists have also been proficient in treatment for allergy.^33,34^ However, the development of antihistamines that target these receptors in the attempt of migraine treatment provide mixed results in their effectiveness, suggesting the existence of other underlying mechanisms of MC degranulation which remain to be identified.^33,34^

### Role of mast cell degranulation in the activation of meningeal nociceptors

The hypothesis that activation of MCs leads to meningeal nociceptor firing was based on the findings from electrophysiological recordings of meningeal afferents. These studies found that intraperitoneal injection of compound 48/80, an agent capable of MC degranulation, could lead to meningeal nociceptor sensitization.^32^ Using pERK as a marker of nociceptor activation, the study examined meningeal nociceptor responses to compound 48/80 induced MC degranulation. About 15 min after 48/80 was administered, the number of nerve fibers that expressed pERK increased four times over, indicating that MCs possess the capability to activate nociceptors.^32^ Specifically noted was an abundance of pERK expression in the nerve fibers located near the degranulated MCs, further demonstrating a close correlation between MC degranulation and nociceptor activation. This study concluded that MCs possess the ability to activate and communicate with meningeal nerve fibers and nociceptors, suggesting a relationship that may induce migraine pain. In addition to dura, pia afferents may also be important to headaches and MCs are distributed in the pia mater. However, the studies of the roles of mast cells in their interactions with pia afferents and migraine are limited and warrant future research.

Regarding the interactions between nociceptors and MCs, there exists a possible scenario where activation of MCs release compounds that subsequently activate nociceptors, which in turn release neuropeptides that further activate and degranulate MCs, creating a positive-feedback loop that continues until MCs are depleted. Moreover, MC- mediated immune cell recruitment can also promote inflammation, recruiting activated macrophages, microglia leading to a further increase in inflammatory compounds, which then leads to or exaggerates migraine and other neurological phenomena.^35^ This topic is explored further in the following section where research regarding a bi-directional relationship between MCs and the trigeminal nerve is discussed.

### Bi-directional communication between mast cells and trigeminal nerves

Studies have shown bi-directional communication between MCs and peripheral afferents may regulate the nociceptive process.^36,37^ Nociceptors that are in close contact with MCs are known to release certain compounds such as the neuropeptides CGRP and SP, which are often co-released and capable of inducing MC activation and degranulation.^38,39^ Alternatively, the trigeminal nerve and its nociceptors can respond to compounds such as serotonin, PGI_2_, and most notably, histamine, which are also released from MCs.^31,32,36,40^ This type of neurogenic inflammation represents a pathophysiological process defined by the release of neuropeptides and the subsequent increase in inflammatory conditions. CGRP, pituitary adenylate cyclase activating peptide-38 (PACAP-38), and neurokinin A, among others, are released from activated peripheral nociceptive nerve terminals, leading to inflammation and activation of immune cells such as MCs. The product of MC degranulation further excites nociceptors, forming a distinct, cyclical relationship. In post-surgical pain conditions, aggregation and activation of MCs at the injury site profoundly influences the subsequent inflammatory response to sensitize nerve terminals. Previous studies demonstrated that stabilization of MCs attenuated post-surgical pain.^41^ Recent developments in experimental migraine models have also provided increasing amounts of evidence suggesting involvement of MCs and these compounds in migraine headaches.^42^ Neuroimaging studies have provided direct evidence that during the headache phase of migraine, CGRP levels are significantly increased, suggesting the involvement of dural neurogenic inflammation in the onset of migraine headaches. Additionally, studies have found that activation of MCs by neuropeptides causes the release of MC mediators, notably histamine, and in turn, MC compounds further elicit the release of neuropeptides as well as recruit other immune cells.^36^

**Figure 1.**
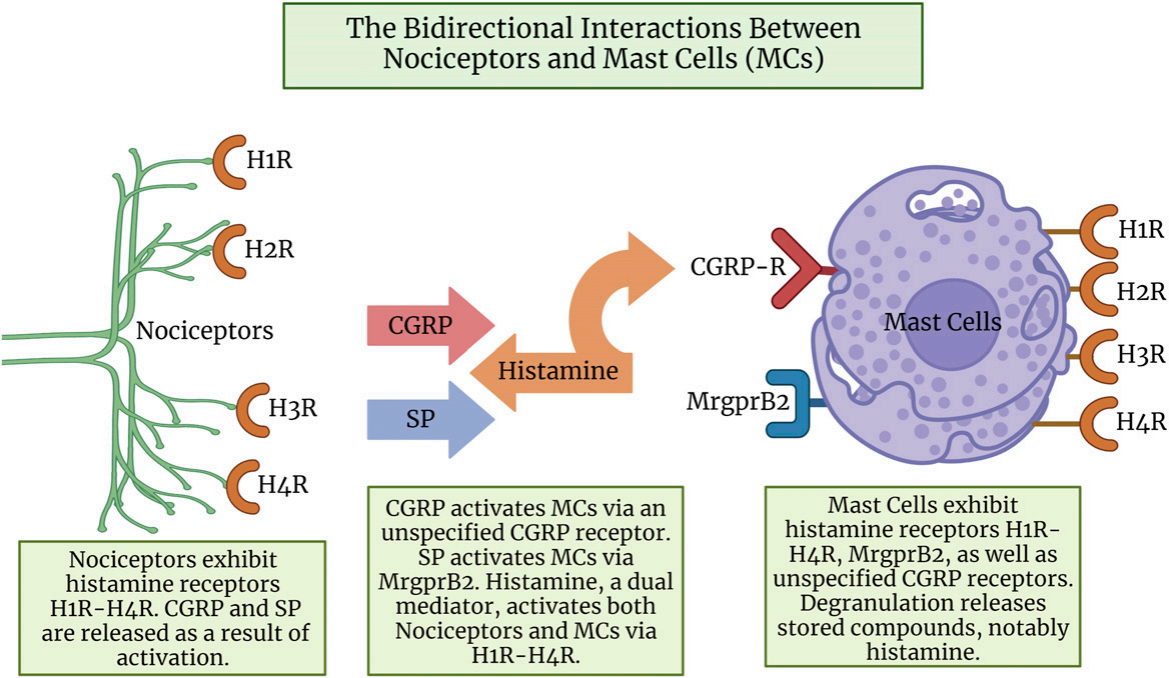
The bidirectional interactions between nociceptors and mast cells (MCs). The peripheral terminals of nociceptors (green) release neuropeptides SP (blue) and CGRP (red) which activate the corresponding receptors located on the MCs (purple). In turn, the MC compound histamine (orange) is released. The dual mediator role of histamine further activates H1-H4 receptors located on both nociceptors and MCs. Since CGRP receptors were not observed in humans but were observed in rats, -R is used. SP: substance P; CGRP: calcitonin-gene-related-peptide; MrgprB2: Mas-Related G-Protein-Coupled Receptor B2; CGRP-R: rat calcitonin-gene-related-peptide receptor. Created with Biorender.com

The opposite sequence occurs when compounds which were released from MC degranulation induce the activation of nociceptors, which release neuropeptides and further activate MCs, establishing a bi-directional relationship (Figure 1). Rosa et al.^36^ described the release of vasoactive intestinal peptide (VIP) which could activate MCs, however, at much weaker potency than CGRP or PACAP-38. A study in patients found that infusion of VIP led to dilation of cranial arteries but could not elicit migraine or headaches.^43^ Recent developments, on the other hand, suggest VIP is relevant in headache,^44^ including a recent small clinical study in which longer infusions of VIP (2 h) resulted in headaches in some patients.^45^ However, due to the recent nature of these VIP migraine studies it was not included in our model.

Pre-clinical evidence involving the administration of CGRP in rats suggested that there may be a link between this neuropeptide and MC activation.^34^ Mobarakeh et al showed that when CGRP was administered in rats, it induced the release of histamine from MCs. Additionally, data provided by Bileviciute et al. demonstrated that intraperitoneal injection of histamine induced the release of CGRP into the cerebrospinal fluid in rats.^36^ Notably as well, Tani et al. showed that when histamine was applied to the nasal mucosa of a guinea pig, CGRP was released from the peripheral terminals of the trigeminal ganglion neurons.^34^ These findings strongly suggest a direct correlation between the presence of histamine and the release of CGRP. Also discussed by Mobarakeh et al. was the MC’s capability to elicit each other’s degranulation as MCs contain histamine receptors. This is described and characterized as dual mediation, where a compound is capable of inducing activation of receptors on other cells while also activating the receptors on its source cell.^36^ The dual mediator function of histamine may also contribute to the activation of H1-H4 receptors and their likely involvement in nociceptive mediation. Implications of histamine and its role in neurogenic inflammation have been examined in the airways, skin, and bladder. However, the review did not specifically evaluate the role of this bi-directional relationship in migraine, but it did indicate the possibility that the bi-directional relationship may also exist in areas other than those mentioned as it describes the existence of this relationship in the skin, airways, and bladder.

Regarding the mechanistic hypothesis of neurogenic inflammation, Ramachandran et al.,^46^ proposed that cortical spreading depression (CSD), a process that results in meningeal inflammation, can lead to MC degranulation and nociceptor sensitization. Either process eventually induces meningeal vasodilation, a characteristic often associated with migraine. Taken together, neurogenic inflammation is a multifaceted process consisting of MCs, sensory nerves, and blood vessels with likely implications for migraine. These findings open up areas for new research revolving around vasodilation and MC degranulation, and the exact roles of different neuropeptides in migraine.

**Figure 2.**
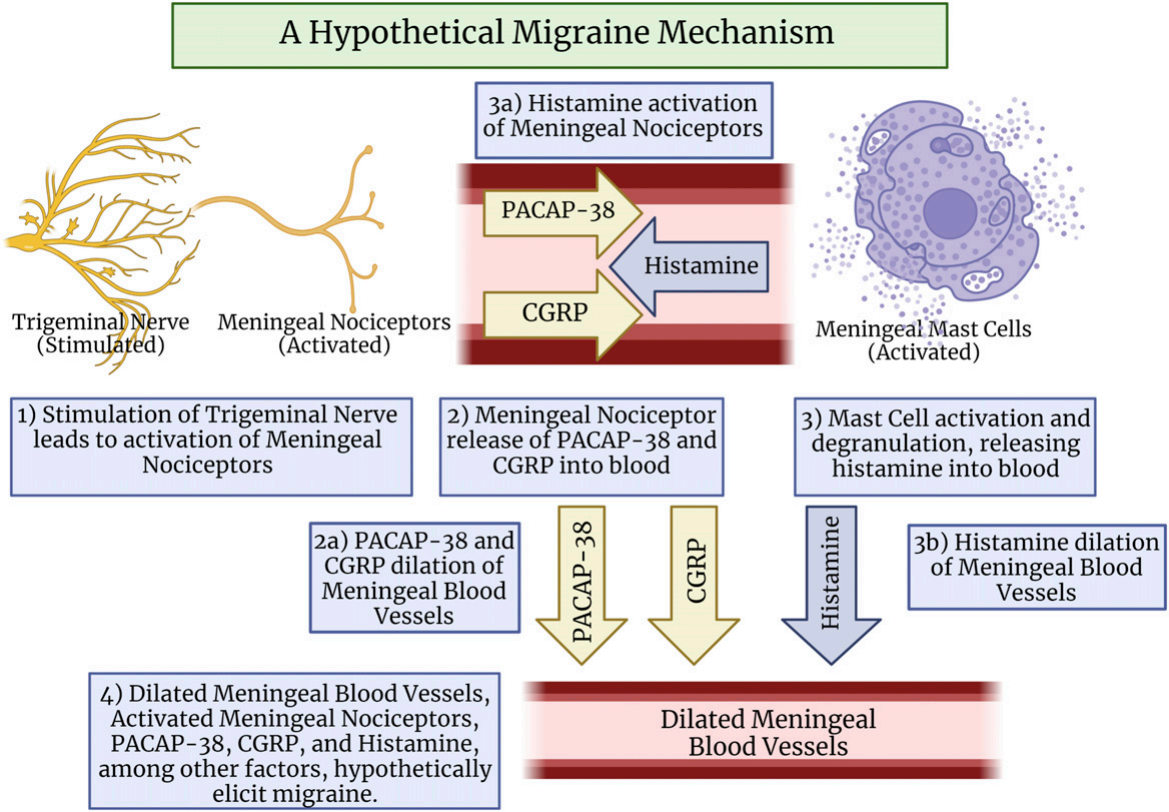
A hypothetical migraine mechanism. Stimulated trigeminal nerves lead to the activation of meningeal nociceptors, releasing neuropeptides PACAP-38 and CGRP from meningeal nociceptors into meningeal blood vessels, contributing to migraine. Both PACAP-38 and CGRP elicit the activation of meningeal mast cells (MCs), which degranulate and release histamine into meningeal blood vessels. Histamine and CGRP contribute to vasodilation in meningeal blood vessels, which may further promote migraine through meningeal nociceptor activation. This mechanism would hypothetically be capable of eliciting migraine headaches. PACAP-38: pituitary adenylate cyclase activating peptide-38; CGRP: calcitonin-gene-related-peptide. Created with Biorender.com

The mediators released from nociceptors and MCs during bidirectional communication, such as CGRP, PACAP-38, VIP, PGI_2_, serotonin, and others, are recognized as compounds that likely contribute to migraine. This suggests that the possible bi-directional relationship between trigeminal meningeal nociceptors and MCs could be capable of evoking the release of a plethora of migraine-inducing mediators which may induce the onset or intensification of migraine.^31,32,42,47^ This prompts the hypothetical mechanism whereby trigeminal nerve stimulation results in the release of neuropeptides CGRP and PACAP-38 from trigeminal meningeal nociceptors, which are compounds understood to be capable of evoking the degranulation of MCs (Figure 2). The degranulation and release of vasoactive molecules, such as histamine, from the meningeal MCs would further elicit migraine pain through the compound’s vasodilating capabilities. Histamine may also intensify and prolong meningeal nociceptor activation through its ability to activate nociceptors,^32,48^ which would then elicit migraine pain.

### Sex difference in migraine prevalence

Prior to the pubescent stage, the prevalence of migraine is similar between boys and girls. However, at the beginning of the pubescent stage, women suffer migraine at a proportion two times more than men.^49^ Overall, women have more prolonged and intense migraine headaches, and higher sensory hypersensitivity in comparison to men. Sex hormones, particularly estrogen, may play a role in this sex difference found in migraine.^49^ A sex difference in migraine has also been demonstrated in animal models by Stucky et al.^50^ utilizing an “inflammatory soup” (IS) of bradykinin, serotonin, histamine, and prostaglandins applied to the dura to elicit migraine like pain. Using this IS model of migraine, they observed pronounced sex differences in migraine-like behaviors and electrophysiological changes, with female rats showing migraine-like effects at much lower dosages and significantly prolonged pain behavioral changes compared to males.^50^ Although this study did not directly examine estrogen as a variable, other studies have shown that increased estrogen levels are elevated in dural afferent fibers and trigeminal sensory neurons.^3^ Furthermore, men who experienced migraine had a higher plasma level of estrogen in comparison to men who did not experience migraine. These findings, together with estrogens ability to directly sensitize trigeminal afferent fibers (particularly through ER_α_),^51^ indicate that elevated estrogen levels play a significant role in sex difference in migraine, most notably through estrogens communication with the trigeminal nerves. Additionally, other hormones such as testosterone, as well as genetic and epigenetic factors may also play a role.^3^

## Future research

### MC specific receptor MrgprB2 and MrgprX2 as new targets for migraine therapeutics

Although MC degranulation via IgE-mediated activation.^24^ is common with regards to allergic reactions, recent developments have identified new receptors on the surface of MCs.^52^ Mrgprs are a family of G-protein-coupled receptors and many (e.g., MrgprA3, C, D) are expressed selectively on primary sensory neurons.^53^ However, MrgprB2 (human orthologue MrgprX2) was identified as a MC-exclusive receptor for basic secretagogues in mice.^54^ Most notably, MrgprB2 is required for the occurrence of neurogenic inflammation and non-histaminergic itch independently of the IgE-FcεRI-histamine axis in mice.^54^ In *MrgprB2-Cre-tdT* mice expressing the tdTomato fluorescent protein (red) under the MrgprB2 promoter, we found the increased aggregation of MCs labeled *in situ* by MrgprB2-Cre-tdT + near the wound after plantar incision. These MCs are critical in recruiting other immune cells to the injury site contributing to thermal and mechanical pain hypersensitivities.^37^ We were then able to connect these two findings by showing that Substance P (SP) activates MrgprB2 receptors causing the release of proinflammatory cytokines and the recruitment of immune cells after tissue injury. Furthermore, SP resulted in immune cell recruitment independent of the Neurokinin 1 receptor (NK1R), implicating MrgprB2 and not NK1R in SP-induced MC degranulation. Furthering the involvement of MrgprB2-mediated signaling in MCs in headache, Compound 48/80, an agonist specific to MrgprB2, has long been used to induce migraine-like pain behavior in rodents, leading to mechanical sensitivity and light aversive behavior.^55,56^ Although MrgprB2 may possess direct involvement in inflammatory pain, additional in vivo studies and further clinical research of human homologue MrgprX2 are warranted to ascertain if it can be an effective new target for migraine pain treatment and future drug development for clinical use.

### Pituitary adenylate cyclase activating peptide-38 (PACAP-38) and MrgprB3 as new targets in future research

Recently, MrgprB3, which is thought to be the rat homologue of human MrgprX2, has also been studied for its involvement in migraine. Multiple pre-clinical studies have implicated PACAP-38 in contributing to migraine pathophysiology^5,57^ and a clinical trial tested targeting PACAP-38 as a possible therapeutic strategy for migraine.^58^ Within the trigeminal vascular system, levels of the vasoactive neuropeptide PACAP-38 has been shown to increase during the acute phase of migraine, and is also elevated in migraineurs during the ictal phase^57^ and intravenous infusion of PACAP-38 can evoke migraine in chronic migraine patients.^59^ However, a recent study from Amgen found that targeting pituitary adenylate cyclase-activating polypeptide type I (PAC1) receptor had no benefit over placebo for migraine prevention.^60^ Initially the PAC1 receptor was thought to be the only receptor activated by PACAP-38, but a recent study showed that it can potently degranulate MCs via the MrgprB3 receptor,^41^ implicating MCs in PACAP-38-induced migraine. PACAP-38 is a far more potent MC activator than PACAP-27, or VIP, and neither may have physiological significance as endogenous MC activators.^41,61^ Additionally, antagonists for PAC1 receptor showed no effect on PACAP-38 activation of MCs.^61^ This further supports the notion that PACAP-induced MC degranulation is not facilitated by the activation of PAC1 receptor but rather through other mechanisms, such as MrgprB3.

A recent episodic cluster headache clinical study found no increase in tryptase from patients infused with PACAP-38.^62^ However, this was a small pilot study whereby blood was collected from the antecubital vein and not from cranial veins, possibly missing released plasma content with a short half-life in the dura mater. Furthermore, this study focused on cluster headache patients, and further study is needed to address migraine patients. Moreover, our lab has uncovered that MrgprB2-activated MCs are capable of releasing multiple inflammatory mediators beyond tryptase. Thus, further study is needed to understand the release time course and expression pattern of cytokines in MrgprX2 + MCs after PACAP-38 treatment. This study does, however, encourage future research over the relevance of PACAP-38 and Mrgprs in migraine.

### CGRP and mast cells remain a future area of research

The recent FDA approval of anti-CGRP antibodies and ongoing clinical trials targeting PACAP-38 for the treatment of migraine lend further support to the contribution of neurogenic inflammation in migraine pathophysiology.^58,63^ Two classes of monoclonal antibodies (mAB) targeting CGRP or its receptor have being used in the preventative treatment of migraine.^64^ A recent clinical trial for the monoclonal CGRP antibody fremanezumab saw on average a reduction of 3.5 migraine days per month with monthly injections compared to placebo and was effective in patients who had failed other treatments.^65^ However, studies investigating the role of CGRP in dural MC activation have yet to find a link. Examination of CGRP and its respective receptors, calcitonin receptor-like receptor (CLR) and receptor activity-modifying protein 1 (RAMP1), in rat dura and human dural vessels observed colocalization of CLR and RAMP1 with tryptase, a marker for MC degranulation.^66^ However, the authors noted that expression of CLR or RAMP1 was not observed in human dural MCs, and thus the existence of human MC-CGRP receptor complex remains unclear. This observation is also in agreement with other studies that found CGRP had no effect on degranulating rat dural MCs in the glyceryl trinitrate migraine model.^67^ Future research aiming to uncover the relationship between CGRP and MCs in other animal models remains warranted.

### Migraine treatment

Current migraine treatment exists in non-pharmacologic and pharmacologic forms with varying specificity. Non-pharmacologic treatment aims to modify patient’s behavioral habits, such as sleep, exercise, and mealtime, all of which have been observed to correlate with migraine evolution.^7^ Pharmacologic treatment of acute migraine is categorized into specific, non-specific, and adjuvant treatments. Treatments range based on the frequency, degree of pain, time of day, and/or other characteristics of migraine, starting from typical non-steroidal anti-inflammatory drugs, such as acetaminophen and ibuprofen, to prescription triptans and other specific treatment drugs.^68^ Recent developments regarding Mrgprs outined in this review add additional dimension to migraine disease discussions, and may be a promising area of future research. In particular, MrgprB2 (human homologue MrgprX2) may be a promising target for migraine treatment. Additionally, the ambiguity around MC-CGRP activation mechanisms provides an area of further research to identify MC-CGRP receptors as a new target for treatment.

## Summary

The large burden to patients and significant social impact of migraine pain have made the study of its origin and management an important area of research. The current hypothesized mechanisms for migraine suggest the involvement of a complex combination of central and peripheral components of the trigeminal nerve, vasodilation, neurogenic inflammation, among other factors. MCs are innate resident immune cells widely distributed in the body and are involved in both inflammatory and allergic diseases. The results of an increasing number of studies have suggested that trigeminal nerve nociceptors and MCs have the ability to mediate each other’s function, making this an innovative area for future research. With the involvement of vasodilation, neurogenic inflammation, MC degranulation, and various neuropeptides in migraine onset, MCs and the trigeminal nerve have been observed to hold an influential role in migraine pathophysiology. However, research regarding the specific relationship between trigeminal nerve nociceptors and meningeal MCs and their possible multidirectional relationship in migraine remains understudied and requires direct evidence in order to support this hypothesis.

## References

[1] Saylor D Steiner TJ . The global burden of headache. Seminars in Neurology 2018; 38(2): 182–190. 29791944 10.1055/s-0038-1646946

[2] Hansen JM Charles A . Differences in treatment response between migraine with aura and migraine without aura: lessons from clinical practice and RCTs. The Journal of Headache and Pain 2019; 20(1): 96. 31492106 10.1186/s10194-019-1046-4 PMC6734209

[3] Gazerani P Cairns BE . Sex-specific pharmacotherapy for migraine: a narrative review. Front Neurosci 2020; 14: 222. 32265634 10.3389/fnins.2020.00222 PMC7101090

[4] Malhotra R . Understanding migraine: potential role of neurogenic inflammation. Annals of Indian Academy of Neurology 2016; 19(2): 175–182. 27293326 10.4103/0972-2327.182302 PMC4888678

[5] Hansen JM Hauge AW Olesen J Ashina M . Calcitonin gene-related peptide triggers migraine-like attacks in patients with migraine with aura. Cephalalgia 2010; 30(10): 1179–1186. 20855363 10.1177/0333102410368444

[6] Burstein R Noseda R Borsook D . Migraine: multiple processes, complex pathophysiology. J Neurosci 2015; 35(17): 6619–6629. 25926442 10.1523/JNEUROSCI.0373-15.2015 PMC4412887

[7] Feniuk W. Humphrey PP Perren MJ Connor HE Whalley ET . Rationale for the use of 5-HT1-like agonists in the treatment of migraine. J Neurol 1991; 238(Suppl 1): S57–S61. 1646289 10.1007/BF01642908

[8] Barbanti P. Aurilia C Fofi L Egeo G Ferroni P . The role of anti-CGRP antibodies in the pathophysiology of primary headaches. Neurological Sciences: Official Journal of the Italian Neurological Society and of the Italian Society of Clinical Neurophysiology 2017; 38(Suppl 1): 31–35. 28527063 10.1007/s10072-017-2907-8

[9] Levy D Burstein R Strassman AM . Calcitonin gene-related peptide does not excite or sensitize meningeal nociceptors: implications for the pathophysiology of migraine. Ann Neurol 2005; 58(5): 698–705. 16240341 10.1002/ana.20619

[10] Khan S Amin FM Christensen CE Ghanizada H Younis S Olinger ACR de Koning PJH Larsson HBW Ashina M . Meningeal contribution to migraine pain: a magnetic resonance angiography study. Brain 2019; 142(1): 93–102. 30590467 10.1093/brain/awy300

[11] Price S Daly DT . Neuroanatomy, trigeminal nucleus. Tampa, FL: StatPearls, 2022. 30969645

[12] Messlinger K Russo AF . Current understanding of trigeminal ganglion structure and function in headache. Cephalalgia 2019; 39(13): 1661–1674. 29989427 10.1177/0333102418786261 PMC7007999

[13] Goto T Iwai H Kuramoto E Yamanaka A . Neuropeptides and ATP signaling in the trigeminal ganglion. Japanese Dental Science Review 2017; 53(4): 117–124. 29201256 10.1016/j.jdsr.2017.01.003 PMC5703691

[14] Messlinger K . The big CGRP flood - sources, sinks and signalling sites in the trigeminovascular system. The Journal of Headache and Pain 2018; 19(1): 22. 29532195 10.1186/s10194-018-0848-0 PMC5847494

[15] Pietrobon D Moskowitz MA . Pathophysiology of migraine. Ann Rev Physiol 2013; 75: 365–391. 23190076 10.1146/annurev-physiol-030212-183717

[16] Iyengar S Johnson KW Ossipov MH Aurora SK . CGRP and the Trigeminal System in Migraine. Headache: The Journal of Head and Face Pain 2019; 59(5): 659–681. 10.1111/head.13529 PMC6593989 30982963

[17] Krystel-Whittemore M Dileepan KN Wood JG . Mast cell: a multi-functional master cell. Front Immun 2015; 6: 620. 10.3389/fimmu.2015.00620 PMC4701915 26779180

[18] Nguyen AV Soulika AM . The dynamics of the skin’s immune system. Inter J Mole Sci 2019; 20(8). 10.3390/ijms20081811 PMC6515324 31013709

[19] Elieh Ali Komi D Wöhrl S Bielory L . Mast cell biology at molecular level: a comprehensive review. Clinical Reviews in Allergy & Immunology 2020; 58(3): 342–365. 31828527 10.1007/s12016-019-08769-2

[20] Malaviya R Ikeda T Ross E Abraham SN . Mast cell modulation of neutrophil influx and bacterial clearance at sites of infection through TNF-α. Nature 1996; 381(6577): 77–80. 8609993 10.1038/381077a0

[21] Theoharides TC Kempuraj D Tagen M Conti P Kalogeromitros D . Differential release of mast cell mediators and the pathogenesis of inflammation. Immunolo Rev 2007; 217: 65–78. 10.1111/j.1600-065X.2007.00519.x 17498052

[22] Metcalfe DD Baram D Mekori YA . Mast cells. Physiolo Rev 1997; 77(4): 1033–1079. 10.1152/physrev.1997.77.4.1033 9354811

[23] Wernersson S Pejler G . Mast cell secretory granules: armed for battle. Nature Rev Immun 2014; 14(7): 478–494. 10.1038/nri3690 24903914

[24] Choi HW Abraham SN . In Vitro and In Vivo IgE-/Antigen-Mediated mast cell activation*.* Methods in Molecular Biology, 2018; 1799: 71–80. 29956145 10.1007/978-1-4939-7896-0_7

[25] Bayar Muluk N Bafaqeeh SA Cingi C . Anti-IgE treatment in allergic rhinitis. International Journal of Pediatric Otorhinolaryngology 2019; 127: 109674. 31526939 10.1016/j.ijporl.2019.109674

[26] Wang F Yang TB Kim BS . The return of the mast cell: new roles in neuroimmune itch biology. Journal of Investigative Dermatology 2020; 140(5): 945–951. 32248995 10.1016/j.jid.2019.12.011 PMC7183871

[27] Galli SJ Kalesnikoff J Grimbaldeston MA Piliponsky AM Williams CMM Tsai M . Mast cells as “tunable” effector and immunoregulatory cells: recent advances. Ann Rev Immun 2005; 23: 749–786. 15771585 10.1146/annurev.immunol.21.120601.141025

[28] da Silva EZ Jamur MC Oliver C . Mast cell function. Journal of Histochemistry & Cytochemistry 2014; 62(10): 698–738. 25062998 10.1369/0022155414545334 PMC4230976

[29] Heatley RV Denburg JA Bayer N Bienenstock J . Increased plasma histamine levels in migraine patients. Clinical & Experimental Allergy 1982; 12(2): 145–149. 10.1111/j.1365-2222.1982.tb01633.x 6176358

[30] Sarchielli P Alberti A Baldi A Coppola F Rossi C Pierguidi L Floridi A Calabresi P . Proinflammatory cytokines, adhesion molecules, and lymphocyte integrin expression in the internal jugular blood of migraine patients without aura assessed ictally. Headache: The Journal of Head and Face Pain 2006; 46(2): 200–207. 10.1111/j.1526-4610.2006.00337.x 16492228

[31] Zhang XC Strassman AM Burstein R Levy D . Sensitization and activation of intracranial meningeal nociceptors by mast cell mediators. Journal of Pharmacology and Experimental Therapeutics 2007; 322(2): 806–812. 17483291 10.1124/jpet.107.123745

[32] Levy D Burstein R Kainz V Jakubowski M Strassman AM . Mast cell degranulation activates a pain pathway underlying migraine headache. Pain 2007; 130(1–2): 166–176. 17459586 10.1016/j.pain.2007.03.012 PMC2045157

[33] Yuan H Silberstein SD . Histamine and migraine. Headache: The Journal of Head and Face Pain 2018; 58(1): 184–193. 10.1111/head.13164 28862769

[34] Worm J Falkenberg K Olesen J . Histamine and migraine revisited: mechanisms and possible drug targets. The Journal of Headache and Pain 2019; 20(1): 30. 30909864 10.1186/s10194-019-0984-1 PMC6734463

[35] Conti P D’Ovidio C Conti C Gallenga CE Lauritano D Caraffa A Kritas SK Ronconi G . Progression in migraine: role of mast cells and pro-inflammatory and anti-inflammatory cytokines. Eur J Pharmacol 2019; 844: 87–94. 30529470 10.1016/j.ejphar.2018.12.004

[36] Rosa AC Fantozzi R . The role of histamine in neurogenic inflammation. British Journal of Pharmacology 2013; 170(1): 38–45. 23734637 10.1111/bph.12266 PMC3764847

[37] Green DP Limjunyawong N Gour N Pundir P Dong X . A mast-cell-specific receptor mediates neurogenic inflammation and pain. Neuron 2019; 101(3): 412–420. 30686732 10.1016/j.neuron.2019.01.012 PMC6462816

[38] Yu Y Blokhuis BR Garssen J Redegeld FA . Non-IgE mediated mast cell activation. European Journal of Pharmacology 2016; 778: 33–43. 26164792 10.1016/j.ejphar.2015.07.017

[39] Raddant AC Russo AF . Calcitonin gene-related peptide in migraine: intersection of peripheral inflammation and central modulation. Expert Reviews in Molecular Medicine 2011; 13: e36. 22123247 10.1017/S1462399411002067 PMC3383830

[40] Zeitz KP Guy N Malmberg AB Dirajlal S Martin WJ Sun L Bonhaus DW Stucky CL Julius D Basbaum AI . The 5-HT3Subtype of serotonin receptor contributes to nociceptive processing via a novel subset of myelinated and unmyelinated nociceptors. J Neurosci 2002; 22(3): 1010–1019. 11826129 10.1523/JNEUROSCI.22-03-01010.2002 PMC6758503

[41] Pedersen SH la Cour SH Calloe K Hauser F Olesen J Klaerke DA Jansen-Olesen I . PACAP-38 and PACAP(6-38) Degranulate Rat Meningeal Mast Cells via the Orphan MrgB3-Receptor. Front Cell Neurosci 2019; 13: 114. 30983973 10.3389/fncel.2019.00114 PMC6447718

[42] Ramachandran R . Neurogenic inflammation and its role in migraine. Semi Immunopathol 2018; 40(3): 301–314. 10.1007/s00281-018-0676-y 29568973

[43] Rahmann A Wienecke T Hansen J Fahrenkrug J Olesen J Ashina M . Vasoactive intestinal peptide causes marked cephalic vasodilation, but does not induce migraine. Cephalalgia 2008; 28(3): 226–236. 18254893 10.1111/j.1468-2982.2007.01497.x

[44] Waschek JA Baca SM Akerman S . PACAP and migraine headache: immunomodulation of neural circuits in autonomic ganglia and brain parenchyma. The Journal of Headache and Pain 2018; 19(1): 23. 29536279 10.1186/s10194-018-0850-6 PMC5849772

[45] Pellesi L Al-Karagholi MA De Icco R Coskun H Elbahi FA Lopez-Lopez C Snellman J Hannibal J Amin FM Ashina M . Effect of Vasoactive Intestinal Polypeptide on Development of Migraine Headaches. JAMA Network Open 2021; 4(8): e2118543. 34357396 10.1001/jamanetworkopen.2021.18543 PMC8346940

[46] Ramachandran R Bhatt DK Ploug KB Hay-Schmidt A Jansen-Olesen I Gupta S Olesen J . Nitric oxide synthase, calcitonin gene-related peptide and NK-1 receptor mechanisms are involved in GTN-induced neuronal activation. Cephalalgia 2014; 34(2): 136–147. 24000375 10.1177/0333102413502735

[47] Spekker E Tanaka M Szabó Á Vécsei L . Neurogenic inflammation: the participant in migraine and recent advancements in translational research. Biomedicines 2021; 10(1). 10.3390/biomedicines10010076 PMC8773152 35052756

[48] Alstadhaug KB . Histamine in migraine and brain. Headache: The Journal of Head and Face Pain 2014; 54(2): 246–259. 10.1111/head.12293 24433203

[49] Wilcox SL Ludwick AM Lebel A Borsook D . Age- and sex-related differences in the presentation of paediatric migraine: a retrospective cohort study. Cephalalgia 2018; 38(6): 1107–1118. 28766966 10.1177/0333102417722570

[50] Stucky NL Gregory E Winter MK He Y-Y Hamilton ES McCarson KE Berman NEJ . Sex differences in behavior and expression of CGRP-related genes in a rodent model of chronic migraine. Headache: The Journal of Head and Face Pain 2011; 51(5): 674–692. 10.1111/j.1526-4610.2011.01882.x PMC4079043 21521205

[51] Rowan MP Berg KA Roberts JL Hargreaves KM Clarke WP . Activation of Estrogen Receptor α Enhances Bradykinin Signaling in Peripheral Sensory Neurons of Female Rats. J Pharmacolo Exper Therapeut 2014; 349(3): 526–532. 10.1124/jpet.114.212977 PMC4019325 24706985

[52] Green DP . The role of Mrgprs in pain. Neurosci Lett 2021; 744: 135544. 33421487 10.1016/j.neulet.2020.135544

[53] Liu Q Dong X . The role of the Mrgpr receptor family in itch. Pharmacology of Itch 2015; 226: 71–88. 10.1007/978-3-662-44605-8_5 25861775

[54] McNeil BD Pundir P Meeker S Han L Undem BJ Kulka M Dong X . Identification of a mast-cell-specific receptor crucial for pseudo-allergic drug reactions. Nature 2015; 519(7542): 237–241. 25517090 10.1038/nature14022 PMC4359082

[55] Sicuteri F . Mast cells and their active substances: their role in the pathogenesis of migraine. Headache: The Journal of Head and Face Pain 1963; 3: 86–92. 10.1111/j.1526-4610.1963.hed0303086.x 14094109

[56] Ramachandran R Wang Z Saavedra C DiNardo A Corr M Powell SB Yaksh TL . Role of Toll-like receptor 4 signaling in mast cell-mediated migraine pain pathway. Molecular Pain 2019; 15: 1744806919867842. 31342858 10.1177/1744806919867842 PMC6688145

[57] Tuka B Helyes Z Markovics A Bagoly T Szolcsányi J Szabó N Tóth E Kincses ZT Vécsei L Tajti J . Alterations in PACAP-38-like immunoreactivity in the plasma during ictal and interictal periods of migraine patients. Cephalalgia 2013; 33(13): 1085–1095. 23598374 10.1177/0333102413483931

[58] Rubio-Beltrán E Correnti E Deen M Kamm K Kelderman T Papetti L Vigneri S MaassenVanDenBrink A Edvinsson L . PACAP38 and PAC1 receptor blockade: a new target for headache? The Journal of Headache and Pain 2018; 19(1): 64. 30088106 10.1186/s10194-018-0893-8 PMC6081277

[59] Schytz HW Birk S Wienecke T Kruuse C Olesen J Ashina M . PACAP38 induces migraine-like attacks in patients with migraine without aura. Brain: A Journal of Neurology 2009; 132(Pt 1): 16–25. 19052139 10.1093/brain/awn307

[60] Ashina M Doležil D Bonner JH Zhou L Klatt J Picard H Mikol DD . A phase 2, randomized, double-blind, placebo-controlled trial of AMG 301, a pituitary adenylate cyclase-activating polypeptide PAC1 receptor monoclonal antibody for migraine prevention. Cephalalgia 2021; 41(1): 33–44. 33231489 10.1177/0333102420970889 PMC7786389

[61] Baun M Pedersen MHF Olesen J Jansen-Olesen I . Dural mast cell degranulation is a putative mechanism for headache induced by PACAP-38. Cephalalgia 2012; 32(4): 337–345. 22421901 10.1177/0333102412439354

[62] Pellesi L Chaudhry BA Vollesen ALH Snoer AH Baumann K Skov PS Jensen RH Ashina M . PACAP38- and VIP-induced cluster headache attacks are not associated with changes of plasma CGRP or markers of mast cell activation. Cephalalgia 2021; 42. 10.1177/03331024211056248 34822741

[63] Tepper SJ , Anti-Calcitonin Gene-Related Peptide (CGRP) Therapies: Update on a Previous Review After the American Headache Society 60th Scientific Meeting, San Francisco, June 2018*.* Headache: The Journal of Head and Face Pain, 2018. 58 Suppl 3: p. 276–290. 10.1111/head.13417 30403405

[64] Wattiez A-S Wang M Russo AF . CGRP in animal models of migraine. In: Geppetti P (ed). Calcitonin Gene-Related Peptide (CGRP) Mechanisms: Focus on Migraine. Cham: Springer International Publishing, 2019, pp. 85–107. 10.1007/164_2018_187 PMC7008000 30689086

[65] Ferrari MD Diener HC Ning X Galic M Cohen JM Yang R Mueller M Ahn AH Schwartz YC Grozinski-Wolff M Janka L Ashina M . Fremanezumab versus placebo for migraine prevention in patients with documented failure to up to four migraine preventive medication classes (FOCUS): a randomised, double-blind, placebo-controlled, phase 3b trial. The Lancet 2019; 394(10203): 1030–1040. 10.1016/S0140-6736(19)31946-4 31427046

[66] Eftekhari S Warfvinge K Blixt FW Edvinsson L . Differentiation of nerve fibers storing CGRP and CGRP receptors in the peripheral trigeminovascular system. The Journal of Pain 2013; 14(11): 1289–1303. 23958278 10.1016/j.jpain.2013.03.010

[67] Pedersen SH Ramachandran R Amrutkar DV Petersen S Olesen J Jansen-Olesen I . Mechanisms of glyceryl trinitrate provoked mast cell degranulation. Cephalalgia 2015; 35(14): 1287–1297. 25724914 10.1177/0333102415574846

[68] Aguilar-Shea AL Membrilla MD JA Diaz-de-Teran J . Migraine review for general practice. Atención Primaria 2022; 54(2): 102208. 34798397 10.1016/j.aprim.2021.102208 PMC8605054

